# Supplementary material for: Longitudinal Dynamics of Immune Response in Occupational Populations Post COVID-19 Infection in the Changning District of Shanghai, China
Source: Viruses. 2024 Apr 25;16(5):672. doi: 10.3390/v16050672 (PMC11125686; doi:10.3390/v16050672)
Supplement: Supplementary file 1 [file viruses-16-00672-s001.zip › viruses-2968842-supplementary.pdf]

Supplementary Materials

Table S1 Neutralizing antibody changes at different time points in different populations.

	NAbs[1]				
	3-month	4-month	5-month	6-month	7-month
Total	3.39 (3.17, 3.57)	3.29 (3.06, 3.53)	3.28 (2.99, 3.50)	3.27 (3.05, 3.48)	3.23(3.03, 3.58)
Sex					
Male	3.39 (3.16, 3.57)	3.28 (3.02, 3.50)	3.26 (2.99, 3.46)	3.27 (3.04, 3.44)	3.22 (3.01, 3.56)
Female	3.42 (3.22, 3.58)	3.34 (3.18, 3.55)	3.35 (3.13, 3.54)	3.33 (3.13, 3.53)	3.35 (3.12, 3.58)
Age, years					
<35	3.37 (3.17, 3.58)	3.28 (3.04, 3.54)	3.26 (3.02, 3.53)	3.27 (3.08, 3.57)	3.24 (3.07, 3.59)
≥35	3.39 (3.16, 3.55)	3.33 (3.07, 3.52)	3.29 (2.98, 3.49)	3.31 (3.04, 3.42)	3.22 (3.00, 3.56)
Occupation					
Police officers	3.41 (3.17, 3.59)	3.28 (3.03, 3.51)	3.28 (2.99, 3.53)	3.26 (3.03, 3.59)	3.22 (3.01, 3.57)
Healthcare workers	3.36 (3.16, 3.57)	3.32 (3.09, 3.55)	3.28 (3.06, 3.50)	3.33 (3.05, 3.43)	3.34 (3.07, 3.58)
Symptom					
No	3.39 (3.20, 3.52)	3.28 (3.06, 3.55)	3.28 (2.99, 3.54)	3.33 (3.02, 3.52)	3.28 (3.00, 3.58)
Yes	3.38 (3.16, 3.60)	3.30 (3.09, 3.45)	3.28 (3.11, 3.41)	3.27 (3.12, 3.40)	3.22 (3.11, 3.51)
Underlying diseases					
No	3.39 (3.17, 3,57)	3.29 (3.05, 3.54)	3.29 (2.99, 3.51)	3.30 (3.05, 3.47)	3.22 (3.04, 3.59)
Yes	3.43 (3.19, 3.60)	3.31 (3.08, 3.47)	3.25 (3.00, 3.49)	3.24 (3.05, 3.48)	3.42 (2.98, 3.56)

[1] NAbs, Neutralizing antibodies.

Table S2 IgG antibody changes at different time points in different populations.

	IgG[1]				
	3-month	4-month	5-month	6-month	7-month
Total	2.13 (1.92, 2.35)	2.04 (1.82, 2.28)	2.04 (1.81, 2.26)	1.98 (1.79, 2.21)	1.98 (1.72, 2.18)
Sex					
Male	2.08 (1.90, 2.31)	2.01 (1.79, 2.23)	1.97 (1.78, 2.25)	1.94 (1.79, 2.18)	1.93 (1.72, 2.15)
Female	2.20 (2.05, 2.43)	2.17 (1.98, 2.43)	2.12 (1.92, 2.34)	2.09 (1.83, 2.26)	2.04 (1.74, 2.30)
Age, years					
<35	2.08 (1.89, 2.35)	2.01 (1.77, 2.30)	1.96 (1.78, 2.26)	1.95 (1.74, 2.18)	2.00 (1.72, 2.16)
≥35	2.18 (2.04, 2.31)	2.11 (1.91, 2.27)	2.06 (1.86, 2.25)	2.02 (1.82, 2.24)	2.95 (1.74, 2.20)
Occupation					
Police officers	2.16 (1.90, 2.35)	2.02 (1.80, 2.28)	2.05 (1.78, 2.26)	1.94 (1.77, 2.20)	1.94 (1.73, 2.16)
Healthcare workers	2.10 (1.93, 2.33)	2.06 (1.90, 2.30)	2.03 (1.86, 2.27)	2.04 (1.80, 2.24)	2.01 (1.72, 2.19)
Symptom					
No	2.13 (1.93, 2.37)	2.07 (1.84, 2.38)	2.03 (1.80, 2.32)	2.04 (1.77, 2.25)	2.01 (1.71, 2.22)
Yes	2.13 (1.84, 2.20)	1.99 (1.69, 2.16)	2.05 (1.86, 2.17)	1.94 (1.79, 2.14)	1.94 (1.76, 2.11)
Underlying diseases					
No	2.15 (1.93, 2.35)	2.05 (1.83, 2.27)	2.05 (1.85, 2.26)	2.02 (1.80, 2.19)	1.96 (1.73, 2.16)
Yes	2.04 (1.91, 2.40)	1.92 (1.81, 2.37)	1.88 (1.78, 2.32)	1.80 (1.74, 2.25)	2.03 (1.71, 2.22)

[1] IgG, Immunoglobulin G.

Table S3 Peripheral blood lymphocyte subsets of 66 individuals who have infected with COVID-19 across three follow-ups.

	Median (IQR)				Abnormality rate, %		
	3-month	5-month	7-month	P	3-month	5-month	7-month
CD3+ %[1]	66.04 (61.54, 69.46)	65.68 (60.57, 69.89)	65.44 (61.38, 70.12)	0.916	24.24	22.73	25.76
CD4+ %	34.91 (30.97, 38.84)	33.17 (29.06, 39.83)	34.19 (30.86, 39.44)	0.542	37.88	36.36	33.33
CD8+ %	24.49 (20.12, 28.30)	23.56 (19.38, 28.18)	23.83 (19.97, 28.51)	0.725	6.06	4.55	6.06
CD4+/CD8+ ratio	1.41 (1.18, 1.96)	1.38 (1.06, 1.87)	1.50 (1.12, 1.83)	0.945	33.33	34.85	30.30
B cell %	12.99 (10.39, 16.63)	13.67 (10.80, 18.03)	13.48 (10.54, 17.08)	0.620	33.33	28.79	30.30
NK cell %	15.78 (12.31, 21.78)	15.90 (11.55, 21.59)	16.58 (11.68, 20.85)	0.994	10.61	9.09	10.61
Total T cell count	1008.72 (768.80, 1239.19)	1091.02 (929.79, 1374.67)	1096.05 (804.86, 1297.26)	0.090	28.79	13.64	25.76
Helper T cell count	521.17 (433.67, 649.98)	585.95 (460.07, 690.99)	514.63 (416.74 663.02)	0.138	15.15	4.54	12.12
Cytotoxin T cell count	361.09 (257.64, 535.48)	389.10 (298.40, 580.65)	375.67 (253.73, 518.45)	0.361	43.94	33.33	42.42
B cell count	194.48 (136.08, 260.44)	241.97 (175.42, 280.59)	204.20 (167.42, 269.51)	**0.040**	69.70	50.00	65.15
NK cell count	242.39 (151.32, 343.97)	281.70 (186.47, 367.33)	267.49 (177.64, 350.06)	0.382	45.45	33.33	33.33

[1] Percentage: the percentage of immune cells in whole blood. Count: the absolute count of immune cells in per microliter of blood.

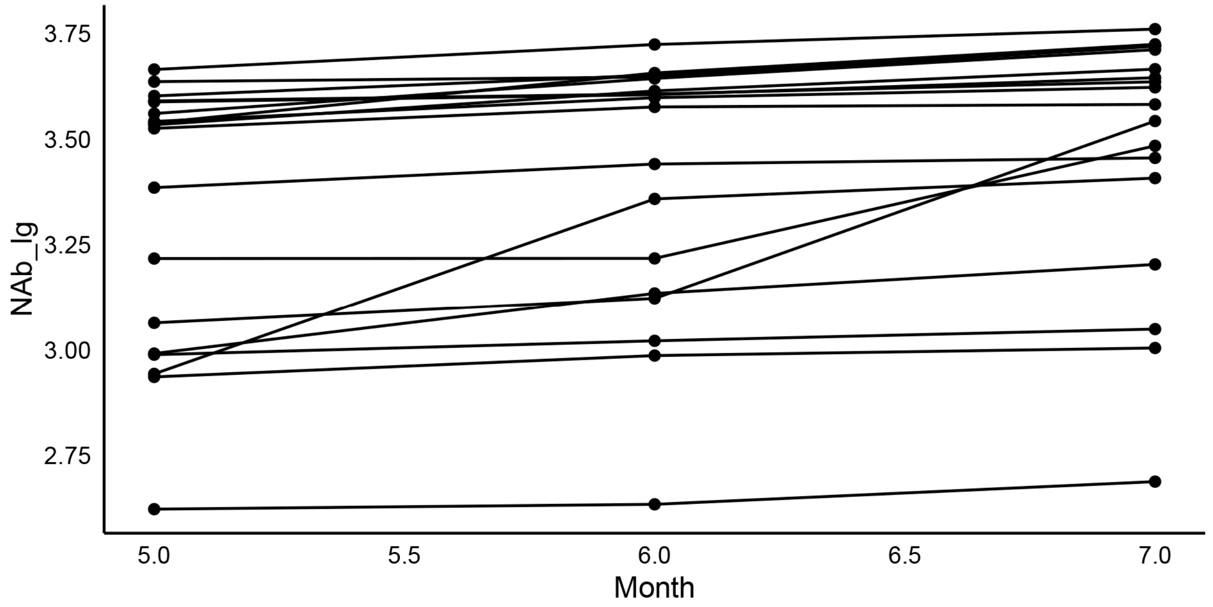

Figure S1. Upward Trend of NAbs Over 5, 6, and 7 months. Lg: logarithmic value to the base 10. NAb, Neutralizing antibody.

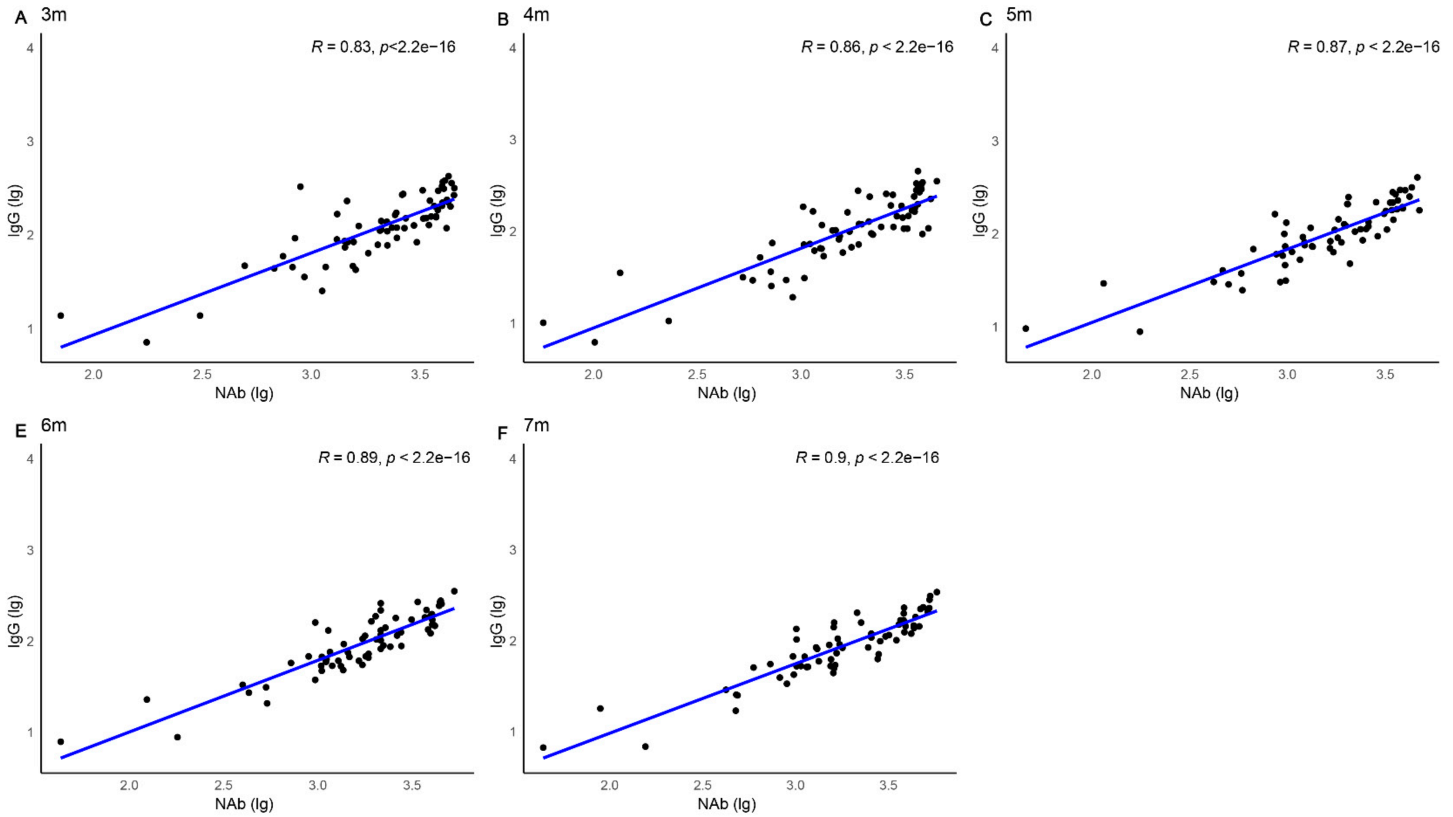

Figure S2. The correlation of NAbs and IgG antibodies across the five follow-up visits. Lg: logarithmic value to the base 10. IgG, Immunoglobulin G. NAb, Neutralizing antibody.
